# Analysis of genomic alternations in epidermal growth factor receptor (EGFR)-T790M-mutated non-small cell lung cancer (NSCLC) patients with acquired resistance to osimertinib therapy

**DOI:** 10.1007/s12094-024-03727-7

**Published:** 2024-09-24

**Authors:** Ping-Chih Hsu, John Wen-Cheng Chang, Li-Chung Chiu, Cheng-Ta Yang, Scott Chih‐Hsi Kuo, Yueh-Fu Fang, Chiao-En Wu

**Affiliations:** 1 Division of Thoracic Oncology, Department of Thoracic Medicine, Chang Gung Memorial Hospital at Linkou, Taoyuan, 33305 Taiwan; 2 Department of Medicine, College of Medicine, Chang Gung University, Taoyuan, 33302 Taiwan; 3 Division of Hematology-Oncology, Department of Internal Medicine, Chang Gung Memorial Hospital at Linkou, Chang Gung University College of Medicine, 5, Fu-Hsing Street, Kwei-Shan, Taoyuan, 33305 Taiwan; 4 Department of Internal Medicine, Taoyuan Chang Gung Memorial Hospital, Taoyuan, 33378 Taiwan; 5 Department of Respiratory Therapy, College of Medicine, Chang Gung University, Taoyuan, 33302 Taiwan

**Keywords:** Epidermal growth factor receptor mutation, Tyrosine kinase inhibitor, Non-small cell lung cancer (NSCLC), T790M, Osimertinib, Acquired resistance

## Abstract

**Background and objectives:**

Genomic alterations after resistance to osimertinib therapy in advanced T790M-mutated non-small cell lung cancer (NSCLC) are complex and poorly understood. In this study, we aimed to detect these genomic alternations via comprehensive next-generation sequencing (NGS) of tissue and liquid biopsies.

**Patients and methods:**

From September 2020 to June 2021, 31 stage IIIB/IV T790M-mutated NSCLC patients who exhibited progressive disease after osimertinib therapy and provided written informed consent were recruited. Liquid and tissue biopsy samples for NGS testing were collected from 31 and 18 patients, respectively. Eighteen study patients had paired NGS data from tissue and liquid biopsies.

**Results:**

With respect to the T790M mutation status, the preservation and loss rates were 33% and 67%, respectively, in both liquid and tissue biopsy samples. Five patients (16.1%) had the C797S mutation (4 liquid samples and 1 tissue sample). Two (6.5%) had MET mutations, 3 (9.7%) had BRAF-V600E mutations, and 1 (3.2%) had a KRAS-G12C mutation. Among the 18 patients who underwent tissue rebiopsies, those with preserved T790M mutation had significantly longer progression-free survival (PFS) with osimertinib therapy than those with T790M mutation loss (10.8 vs. 5.0 months, P = 0.045). Among all patients, those with T790M mutation loss in liquid biopsy samples had longer PFS after osimertinib therapy (10.8 vs. 7.5 months, P = 0.209) and postprogression survival (17.7 vs. 9.6 months, P = 0.132) than those with preserved T790M mutation based on liquid biopsies.

**Conclusions:**

NGS using either tissue or liquid biopsy samples from advanced T790M-mutated NSCLC patients with acquired resistance to osimertinib therapy can detect various genomic alternations. Future studies focusing on subsequent tailored therapies on the basis of NGS results are warranted.

**Supplementary Information:**

The online version contains supplementary material available at 10.1007/s12094-024-03727-7.

## Introduction

Epidermal growth factor receptor (EGFR) and its downstream signaling pathway are common oncogenic pathways in human non-small cell lung cancer (NSCLC). Dysregulation of the EGFR signaling pathway promotes tumorigenesis and cancer progression in NSCLC [1, 2]. The structure of EGFR includes an extracellular receptor as a ligand-binding site, a transmembrane domain, and an intracellular tyrosine protein kinase site [1–3]. The intracellular tyrosine protein kinase site is encoded by exons 18–21, and when genomic mutations occur in exons 18–21, the EGFR downstream prosurvival signaling pathways are activated [1–3]. The threonine-to-methionine substitution point mutation at codon 790 in exon 20 (T790M mutation) has been detected and reported to affect the ATP binding affinity of the EGFR intracellular kinase domain [4, 5]. The T790M mutation mostly appears in EGFR-mutated NSCLC with acquired resistance to first- and second-generation EGFR-tyrosine kinase inhibitors (TKIs) and accounts for the majority of acquired resistance mechanisms to first- and second-generation EGFR-TKIs (40–60%) [4–6]. In a few cases, T790M appears as a primary EGFR mutation in NSCLC (de novo T790M) and leads to unfavorable treatment responses to first- and second-generation EGFR-TKIs [7].

Osimertinib is classified as a third-generation EGFR-TKI and has been shown to effectively inhibit NSCLC with the T790M mutation in preclinical and clinical studies [8]. Osimertinib is the first EGFR-TKI approved by the United States (U.S.) Food and Drug Administration (FDA) for the treatment of T790M-mutated metastatic NSCLC because of its promising efficacy (60% objective response rate (ORR) and 10 months of progression-free survival), as shown in serial AURA trials [8–11]. In the AURA 3 trial, osimertinib was shown to confer a significantly higher ORR and longer PFS than conventional chemotherapy in advanced NSCLC patients with the T790M mutation [11]. Therefore, osimertinib is suggested as a preferred agent for treating advanced NSCLC patients with secondary T790M mutations after they experience acquired resistance to prior 1st- and 2nd-generation EGFR-TKI therapies.

However, most advanced T790M-mutated NSCLCs eventually acquire resistance to osimertinib [12, 13]. The mechanism of acquired resistance to osimertinib in T790M-mutated NSCLC is very complex and poorly understood. In this study, we aimed to analyze genomic alterations in T790M-mutated NSCLC with acquired resistance to osimertinib via next-generation sequencing (NGS) of tissue and liquid biopsy samples. Our study results may impact the sequential treatment strategy for T790M-mutated NSCLC patients with acquired resistance to osimertinib in the future.

## Methods

### Ethics approval

This observational cohort study was approved by the institutional review board (IRB) (no. 202001358B0) of the Chang Gung Medical Foundation. All study subjects signed written informed consent according to the request of the IRB. All study procedures were performed in accordance with the Declaration of Helsinki guidelines. To uphold privacy policies, no identifiable information for the study patients, such as personal birthdays and images, is presented in this manuscript.

### Patients, treatment and follow-up

Between September 2020 and June 2021, a total of 31 stage IIIB/IV lung adenocarcinoma patients harboring both EGFR-activating mutations (exon 19 deletion and L858R) and the EGFR T790M mutation who had progressive disease after osimertinib therapy were recruited. The inclusion criteria were as follows: (1) patients with activating EGFR (exon 19 deletion and L858R) and T790M mutations; (2) patients who received osimertinib for lung cancer treatment; (3) patients with progressive disease to osimertinib therapy; and (4) patients willing to provide informed consent. Patients were excluded for the following reasons: (1) no EGFR T790M mutations; (2) no treatment with osimertinib; and (3) unwillingness to provide informed consent. All study patients received at least computed tomography (CT) as follow-up imaging every 3–4 months to assess the efficacy of treatment. Other additional imaging methods, such as sonograms, fluorodeoxyglucose (FDG)-positron emission tomography (PET), and brain magnetic resonance imaging (MRI), were ordered by clinical physicians to assist in the evaluation of disease status as needed.

The treatment response was assessed via the Response Evaluation Criteria in Solid Tumors (RECIST) version 1.1 and classified as complete response (CR) or partial response (PR). Stable disease (SD) and progressive disease (PD) were considered nonresponses. The length of progression-free survival (PFS) was defined from the first date recorded for osimertinib administration to the first date recorded for imaging PD or death. The length of postprogression survival was defined from the first date of imaging PD after osimertinib therapy to the date of mortality. Overall survival (OS) was defined from the first date recorded for anticancer therapy for lung cancer to the date recorded for a mortality event. The last follow-up time point of this study was 30 September 2022. If patients survived over the last study follow-up time point, survival was censored at the last clinic visit date recorded.

### Detection of EGFR mutations and genetic alternations after resistance to osimertinib

EGFR mutations, including primary and acquired T790M mutations resistant to prior first- or second-generation EGFR-TKIs, were detected via direct sequencing, an amplified refractory mutation system–Scorpion (ARMS/S) or next-generation sequencing (NGS). The NGS methods used to detect genetic alterations after osimertinib resistance in tissue rebiopsy or liquid biopsy samples were proven by GenPan52-TBx (Genconn Biotech, Taiwan) and GenLung12-LBx (Genconn Biotech, Taiwan). NGS of GenLung12-LBx liquid biopsy samples was performed by isolating circulating tumor DNA (ctDNA) from the blood of the study subjects. The genes in the NGS panel are listed in supplementary table S1. All study rebiopsy tissue samples and liquid biopsy blood samples for NGS should be collected after disease progression after osimertinib therapy and before the administration of next-line therapies after osimertinib. Both tissue and liquid biopsy next-generation sequencing (NGS) were performed at a single timepoint neither repeatedly nor serially.

### Statistical analysis

The baseline characteristics of the patients in this study are presented as quantitative variables. PFS and OS were estimated via Kaplan–Meier survival curves. Statistical significance was determined when the two-sided P values were less than 0.05.

## Results

### Baseline characteristics of the study patients

The baseline characteristics of all the study patients are shown in Table 1. All histological types in this study were adenocarcinomas (100%). Twenty-eight patients who acquired T790M mutations and were resistant to prior EGFR-TKI therapy. Afatinib was the most commonly used prior TKI (17 patients, 54.8%), followed by erlotinib (7 patients, 22.6%) and gefitinib (4 patients, 12.9%). Three patients (9.7%) were treatment naïve before osimertinib therapy because of the de novo T790M mutation [7]. Among the 3 patients with de novo T790M mutations, 2 had exon 19 deletions with the T790M mutation, and one had L858R with the T790M mutation. For the osimertinib treatment response, 13 (41.9%) patients achieved a PR, 11 had SD (35.5%), and 7 (22.6%) had PD. All study patients (100%) underwent NGS testing of liquid biopsy samples, and 18 (58.1%) patients received NGS testing of tissue biopsy samples. Eighteen (58.1%) patients had paired NGS testing with both tissue and liquid biopsies.

**Table 1:** Baseline characteristics and clinical information of all study patients

Parameters	No. of cases	%
Gender		
Male	14	45.2
Female	17	54.8
Age (mean ± SD)	60.7 ± 9.5	
Performance status		
0	12	38.7
1	18	58.1
2	1	3.2
Smoking		
Current	3	9.7
Former	7	22.6
Never	19	61.3
Unknown	2	6.4
Morphology		
Adenocarcinoma	31	100
Nonadenocarcinoma	0	
Mutation		
19del	18	58.1
L858R	13	41.9
Stage		
IIIB	4	12.9
IV	27	87.1
Prior TKI regimens		
Gefitinib	4	12.9
Erlotinib	7	22.6
Afatinib	17	54.8
NA	3	9.7
Line		
1 L	3	9.7
2 L	28	90.3
Liver metastasis		
Yes	1	3.2
No	30	96.8
Brain metastasis		
Yes	7	22.6
No	24	77.4
Lung metastasis		
Yes	11	35.5
No	20	64.5
Bone metastasis		
Yes	15	48.4
No	16	51.6
Pleura metastasis		
Yes	13	41.9
No	18	58.1
Adrenal metastasis		
Yes	3	9.7
No	28	90.3
Distant lymph node metastasis		
Yes	4	12.9
No	27	87.1
No. of metastatic sites		
0–1	15	48.4
2–3	13	41.9
4 or more	3	9.7
Osimertinib response		
PR	13	41.9
SD	11	35.5
PD	7	22.6
Sample for NGS		
Tissue biopsy	18	58.1
Liquid biopsy	31	100
Patients with paired tests (Liquid + tissue)	18	58.1

### Genomic alterations after acquired resistance to osimertinib

**Fig. 1:**
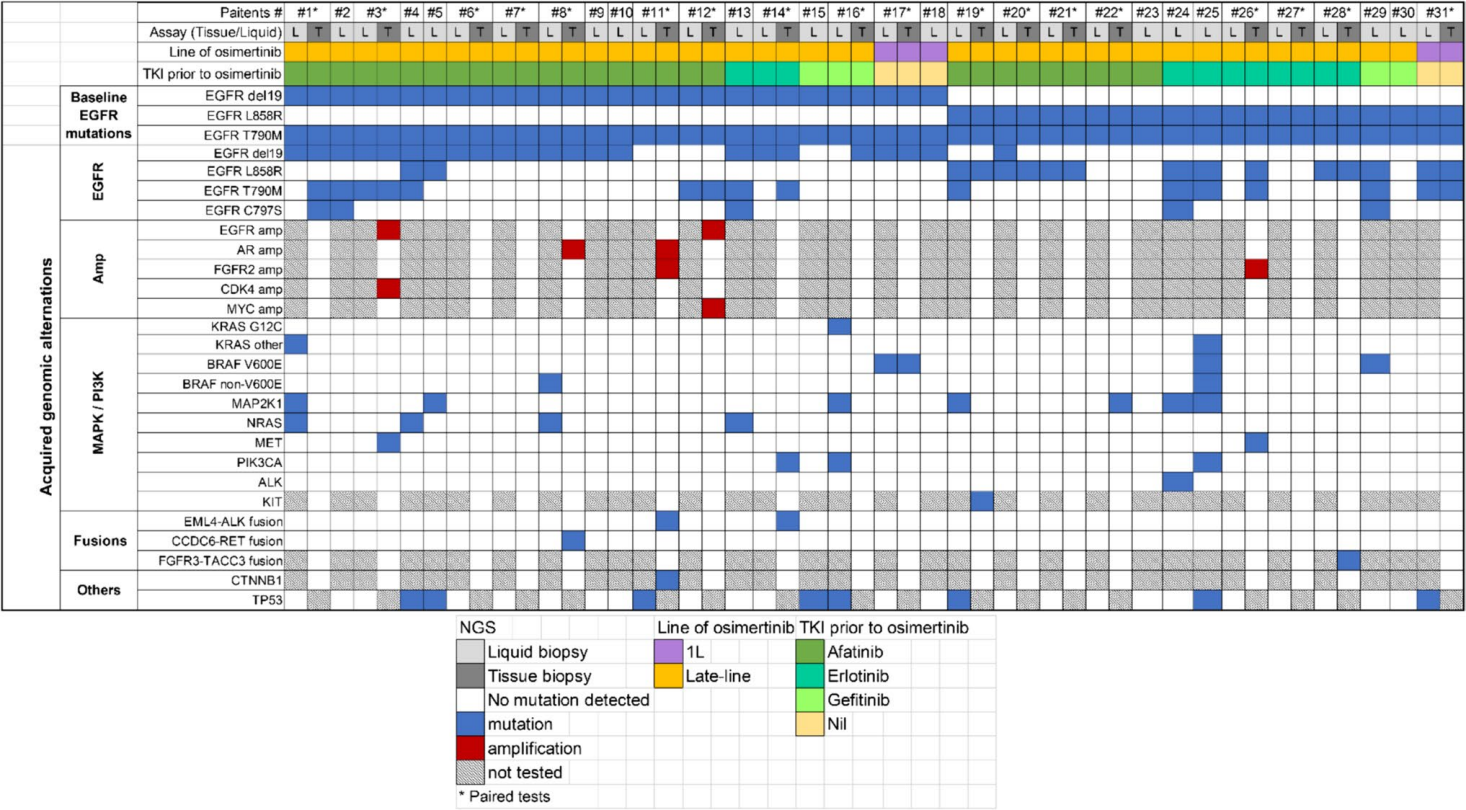
Summary of the genomic alterations in tissue and liquid biopsies from all the study patients

**Fig. 2:**
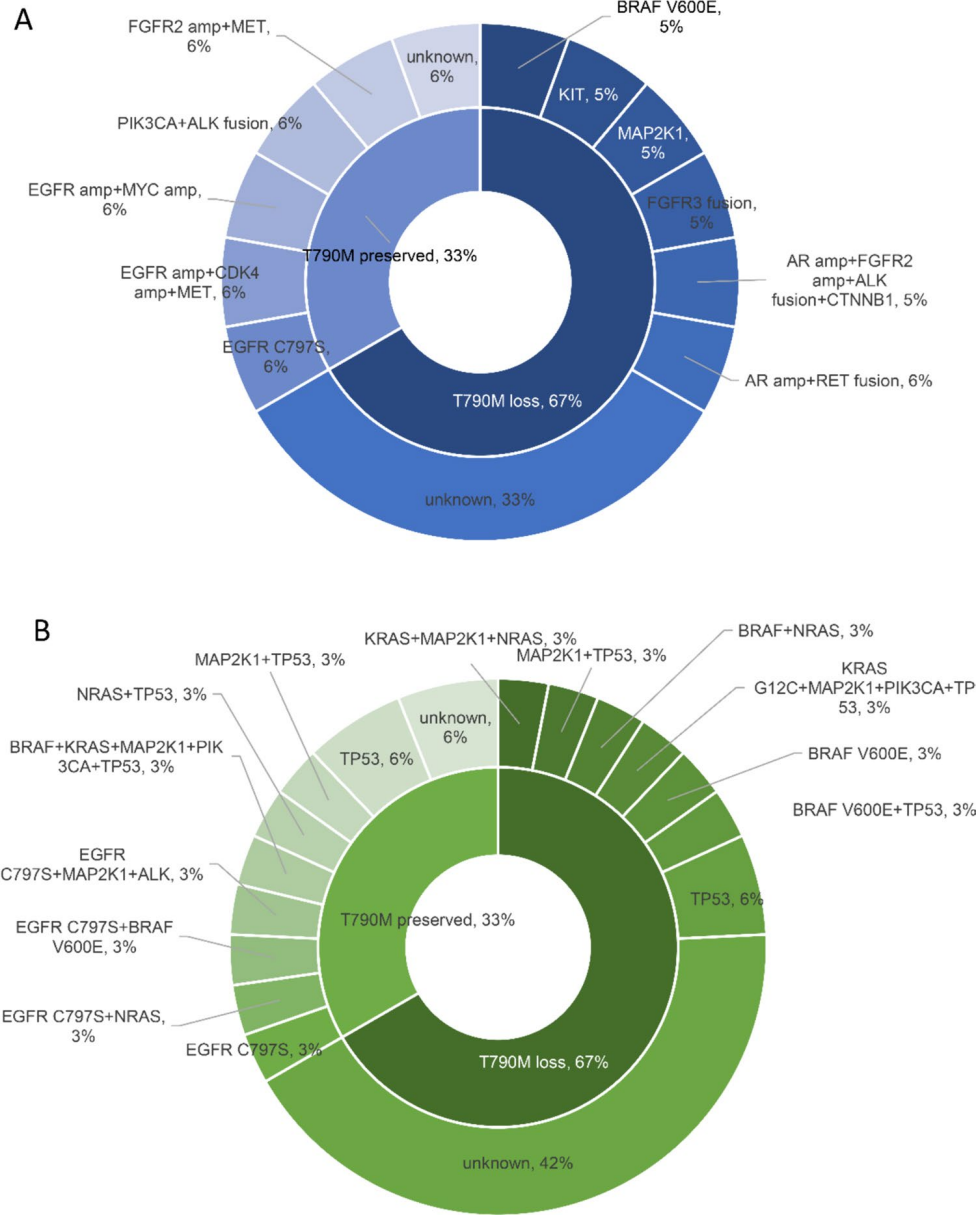
Pie chart of the NGS results of **A** tissue biopsies and **B** liquid biopsies

The genomic alterations assayed by next-generation sequencing (NGS) of liquid and tissue rebiopsy samples after the appearance of osimertinib resistance are shown in Figs. 1 and 2. With respect to the status of the T790M mutation, 6 (33%) patients with tissue NGS data had preserved T790M mutations, and 11 (33%) with liquid biopsy data had preserved T790M mutations (Figs. 1 and 2A, B). Among the 18 patients who underwent tissue NGS testing, 6 (33%) patients did not have additional genetic alterations other than primary EGFR mutations detected (Fig. 2A). Among the 31 patients who underwent liquid biopsy testing, 13 (42%) patients did not have additional genetic alterations other than primary EGFR mutations detected (Fig. 2B). Among the five study patients (16.1%) with EGFR C797S mutations, 4 (12.9%) were detected via liquid biopsy, one (3.2%) was detected via tissue NGS, and the C797S and T790M mutations of all 5 (16.1%) patients were in cis. For the acquired alternations of amplification, no (0%) MET amplification was detected in this study. Regarding the alternations in mutations, 3 (9.7%) patients had KRAS mutations (1 had a KRAS-G12C mutation, and 2 had non-KRAS-G12C mutations), and 5 (16.1%) had BRAF mutations (3 had V600E mutations, and 2 had non-V600E mutations). Two (6.5%) patients had point mutations in MET. With respect to fusion alternations, 2 (6.5%) were EML4-ALK fusions, and 1 (3.2%) was a CCDC6-RET fusion. For other genetic alternations, TP53 was the most frequently detected mutation (10 patients (32.23%)). The patients whose acquired genomic alternations cooccurred with the corresponding approved targeted drugs in this study are summarized in Table 2.

**Table 2:** Information of study patients with co-occurrence of acquired genomic alternations with corresponding approved targeted drugs

Patients	Co-occurrence of acquired genomic alternations	Methods of detection
Patient #3	EGFR exon 19 deletion/T790M + MET mutation	Tissue biopsy
Patient #8	EGFR exon 19 deletion + CCDC6-RET fusion	Tissue biopsy
Patient #14	EGFR exon 19 deletion/T790M + EML4-ALK fusion	Tissue biopsy
Patient #25	EGFR L858R/T790M + BRAF-V600E mutation	Liquid biopsy
Patient #29	EGFR L858R/T790M/C797S + BRAF-V600E mutation	Liquid biopsy

### Clinical outcomes for all study patients

**Fig. 3:**
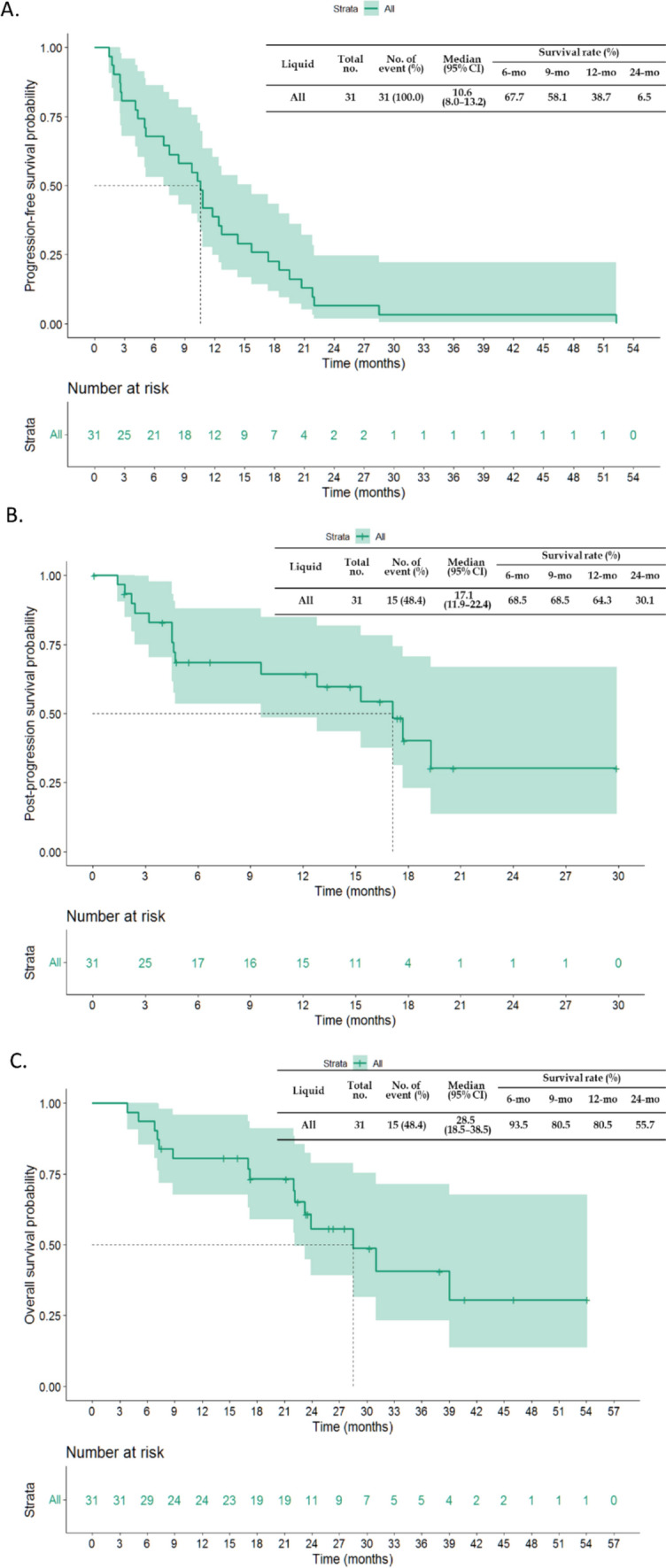
The clinical outcomes of all study patients. **A** The median PFS of patients receiving osimertinib therapy was 10. 6 months (95% CI 8.0–13.2). **B** The median postosimertinib progression survival of all study patients was 17.1 months (95% CI 11.9–22.4). **C** The median OS of all study patients was 28.5 months (95% CI 18.5–38.5)

**Fig. 4:**
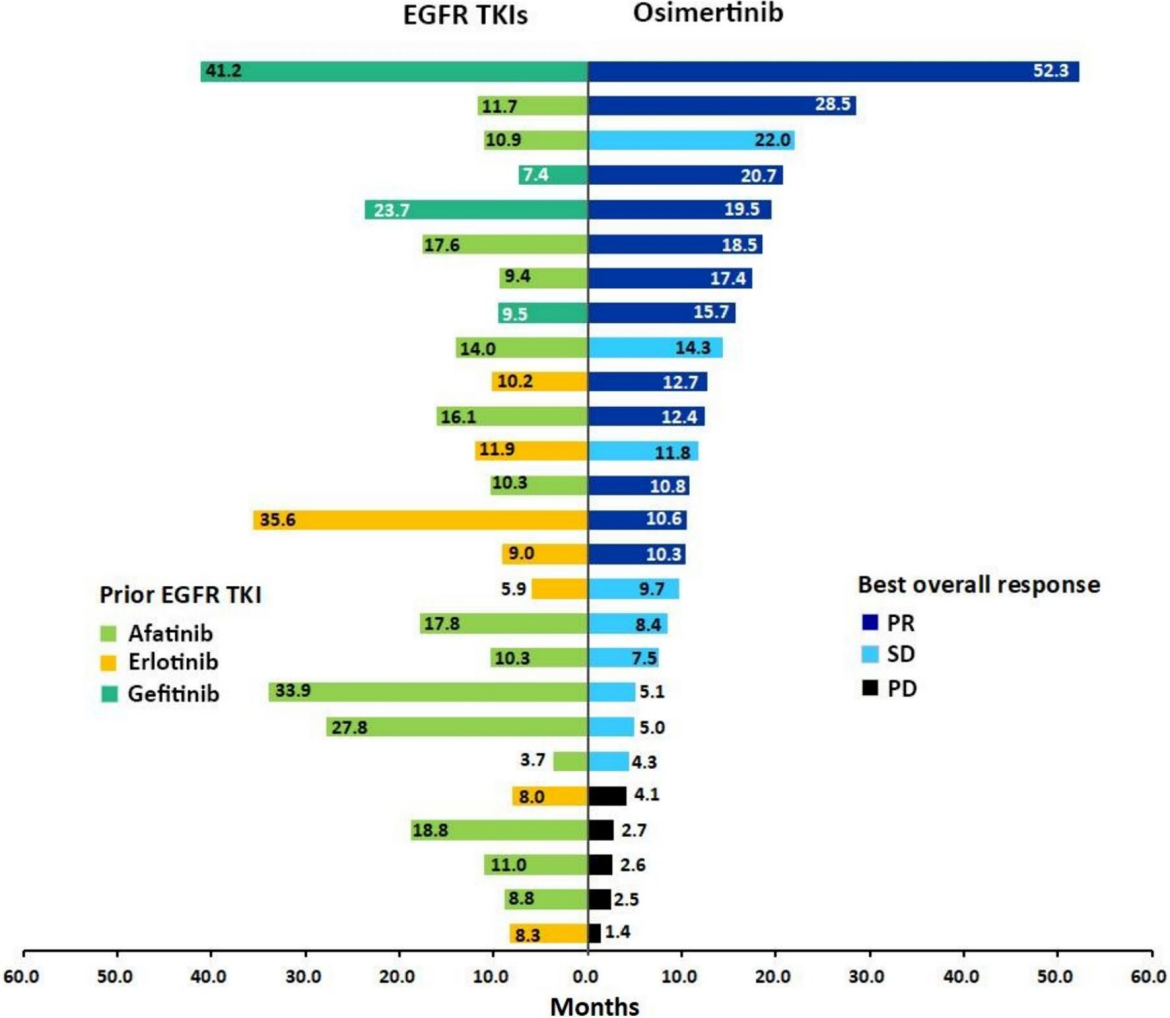
Treatment length of EGFR-TKIs

The median PFS of all the study patients receiving osimertinib therapy was 10.6 months (95% confidence interval (CI), 8.0–13.2) (Fig. 3A). The median postosimertinib progression survival of all study patients was 17.1 months (95% CI 11.9–22.4). The 1 year and 2 year postosimertinib progression survival rates were 64.3% and 30.1%, respectively (Fig. 3B). The median OS of all the study patients was 28.5 months (95% CI 18.5–38.5), and more than half of the study patients survived more than 2 years (55.7%) (Fig. 3C). The durations of prior-line EGFR-TKI therapy and osimertinib treatment in 26 patients are summarized in Fig. 4. Two patients were not listed because they had received at least one chemotherapy regimen between prior EGFR-TKIs and osimertinib.

### Clinical outcomes for different T790M mutation statuses after osimertinib resistance

**Fig. 5:**
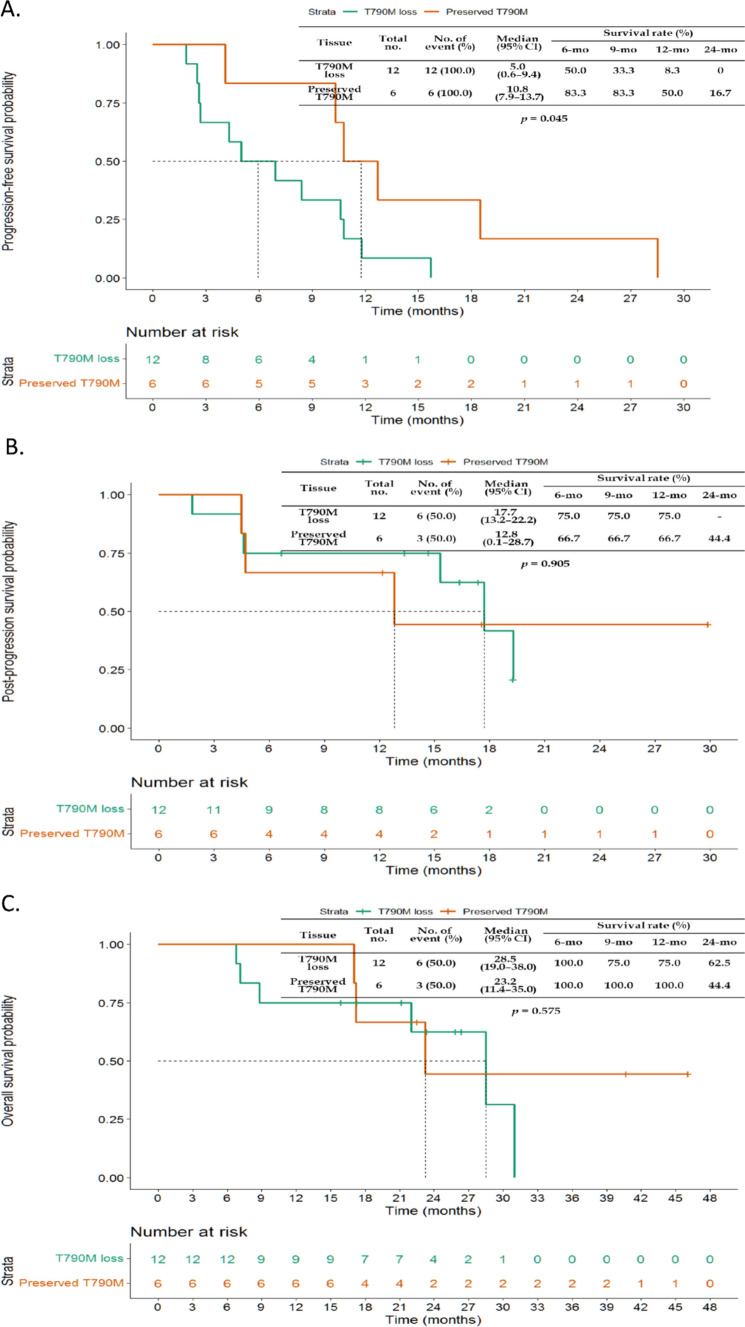
Comparison of clinical outcomes between patients with different postprogression T790M mutation statuses in tissue biopsies. **A** Comparison of the median PFS of osimertinib-treated patients. **B** Comparison of the median postprogression survival. **C** Comparison of the median OS

With respect to the T790M mutation status of tissue NGS results in 18 patients who underwent tissue rebiopsy, preserved T790M mutation patients had significantly longer median PFS after osimertinib therapy than did those with T790M mutation loss (10.8 vs. 5.0 months, P = 0.045) (Fig. 5A). There was no significant difference in postosimertinib progression survival or OS between patients with preserved T790M mutation and those with T790M mutation loss who underwent tissue rebiopsy (Fig. 5B, C).

**Fig. 6:**
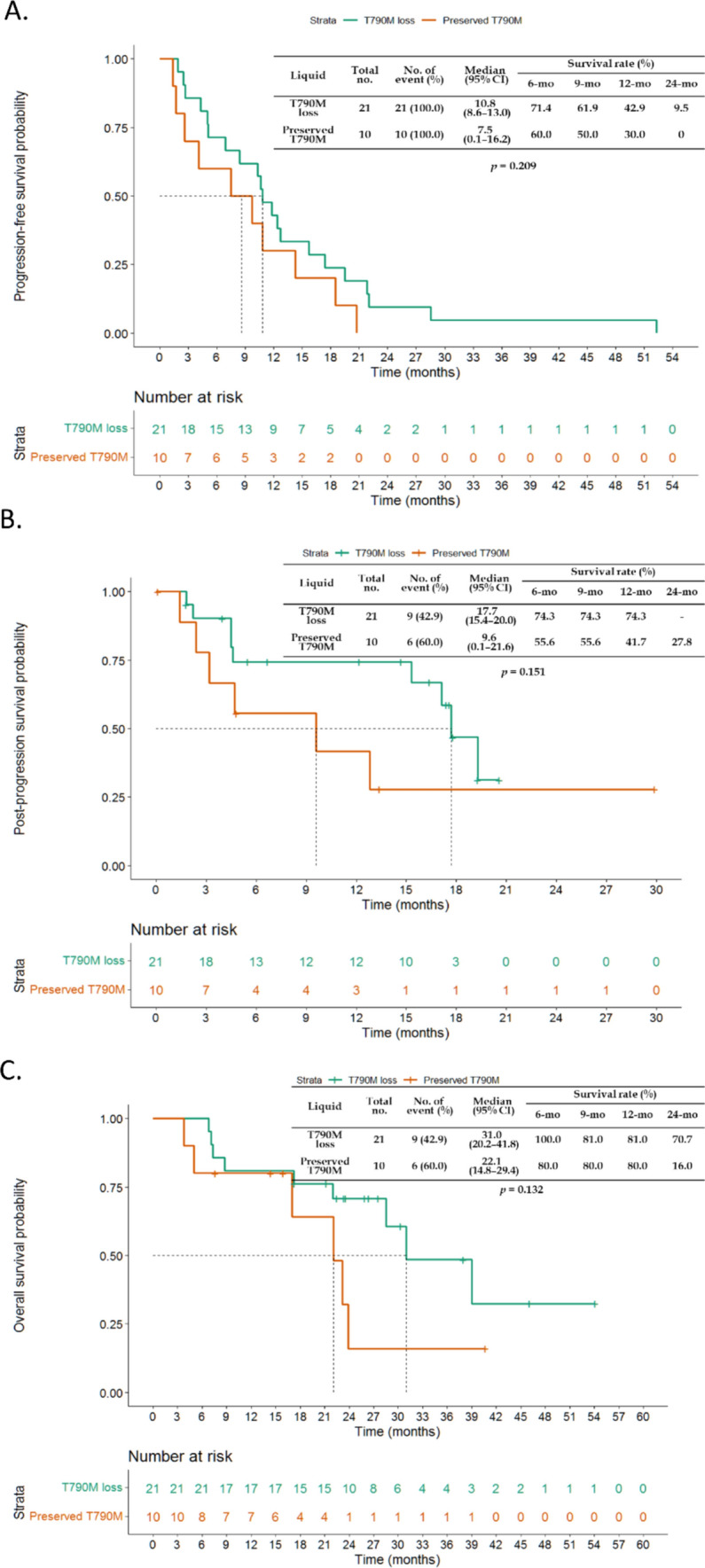
Comparison of clinical outcomes between patients with different postprogression T790M mutation statuses in liquid biopsies. **A** Comparison of the median PFS of osimertinib-treated patients. **B** Comparison of the median postprogression survival. **C** Comparison of the median OS

For the T790M mutation status of liquid biopsy in all 31 study patients, patients with T790M mutation loss had longer PFS after osimertinib therapy than did those with preserved T790M mutation, but no significant difference was observed (10.8 vs. 7.5 months, P = 0.209) (Fig. 6A). Patients with T790M mutation loss had longer postosimertinib progression survival than those with preserved T790M mutation did, but the difference was not significant (17.7 vs. 9.6 months, P = 0.132) (Fig. 6B). There was no significant difference in OS between patients with preserved T790M mutation and those with T790M mutation loss who underwent liquid biopsy (Fig. 6C).

## Discussion

The results of this study provide important clinical insights for patients with T790M-mutated NSCLC with acquired resistance to prior osimertinib therapy. First, we showed the genomic alterations and profiles in T790M-mutated patients with acquired resistance to osimertinib. Second, the preservation or loss of the T790M mutation in either tissue or liquid biopsies had different clinical impacts on prior osimertinib therapy and postprogression survival. In addition, using both liquid and tissue biopsy samples for NGS testing potentially revealed more genomic alterations, and these genomic alteration profiles may help clinical physicians plan treatment strategies for T790M-mutated NSCLC patients with acquired resistance to osimertinib therapy.

With respect to tissue rebiopsy, previous studies have suggested that NGS should be used to assess genetic alterations after acquired resistance to osimertinib because the mechanism of osimertinib resistance is very heterogeneous and complex [12–16]. Two previous studies revealed that 25–32% of T790M-mutated NSCLC patients had preserved T790M mutations after resistance to osimertinib, and the results of this study (33% in both tissue and liquid biopsies) were consistent with these two previous studies [12, 16]. In the same two previous studies, patients with preserved T790M mutation had significantly longer PFS and OS after osimertinib than those with loss of the T790M mutation after resistance [12, 16]. Previous studies have suggested that T790M is the dominant mutation in rebiopsy tumors with preserved T790M mutations and that osimertinib may have prolonged efficacy in tumors with T790M-dominant mutations. In contrast, rebiopsy of tumors with loss of the T790M mutation suggested increased intratumoral genomic heterogeneity, and subclones with genetic alternations other than T790M may be selected and become dominant mutations after osimertinib therapy. Therefore, the selected non-T790M subclones may lead to a resistance mechanism to osimertinib treatment [17, 18].

With respect to liquid biopsy, in this study, patients who had preserved T790M mutation in liquid biopsy after osimertinib resistance tended to have shorter PFS with osimertinib and postprogression survival than those with loss of the T790M mutation in liquid biopsy. Several previous studies have investigated the correlation between the efficacy of osimertinib and the EGFR-mutated circulating tumor (ct) DNA level in T790M-mutated patients receiving osimertinib therapy. These studies reported that patients with loss of the T790M mutation in ctDNA had significantly longer PFS and OS after osimertinib therapy than those with maintenance of T790M ctDNA [5, 19–21]. The results of our study were compatible with those of these previous studies, but statistical significance was not achieved in our study because of the small study population (n = 31). Previous studies have reported that plasma ctDNA levels are correlated with tumor and metastatic burdens and that the loss of detectable ctDNA early in EGFR-TKI treatment is associated with favorable clinical outcomes [22, 23]. Taken together, the detection of maintained T790M ctDNA in liquid biopsy samples was found to be a factor associated with unfavorable clinical outcomes in patients receiving osimertinib therapy.

Previous studies encouraged repeated rebiopsy, both liquid and tissue, for NGS testing in patients with advanced EGFR-mutated NSCLC with progressive disease and acquired resistance [24–26]. These previous studies suggested that repeated rebiopsies increased the detection rate of acquired T790M mutations and may allow more advanced EGFR-mutated patients to receive osimertinib therapy after acquired resistance to prior first- or second-generation EGFR-TKIs [24–26]. In this study, all patients received liquid biopsies, whereas 18 (58.1%) patients underwent tissue rebiopsies for NGS testing. Ideally, all patients should receive tissue rebiopsies when possible, but some patients do not receive tissue rebiopsies because of concerns about the safety of invasive procedures or procedure-unapproachable tumor sites, such as deep intrathoracic or intraabdominal tumors beside great vessels [27].

In this study, the C797S mutation was detected in 5 (16.1%) patients (1 by tissue rebiopsy and 4 by liquid biopsy), all of whom cooccurred with preserved T790M mutations in cis, and the acquired C797S mutation rate in our study was in line with that reported in previous studies [12, 13, 16]. The concurrent C797S and T790M mutations are classified into cis and trans forms, and the cis or trans forms of the C797S and T790M mutations respond to different combination treatment strategies [28]. For C797S and T790M mutations in trans, previous studies reported that those acquired from trans C797S and T790M mutations respond to the combination of osimertinib and first-generation EGFR-TKI (gefitinib) therapy [28, 29]. For C797S and T790M mutations in cis, combination therapy with brigatinib and cetuximab has been shown to be more effective in terms of the response rate and PFS than other therapies [30]. In patients with the C797S mutation and loss of the T790M mutation after progression to osimertinib therapy, first-generation EGFR-TKIs (gefitinib and erlotinib) have been reported to be effective in treating NSCLC with the C797S mutation alone [28, 30]. Ideally, the unmet need for advanced NSCLC patients with acquired EGFR C797X mutations after progression to osimertinib is the development of fourth-generation EGFR-TKIs to overcome this resistance. However, several fourth-generation EGFR-TKIs are still under investigation in preclinical studies or early-phase clinical trials [31].

MET amplification is a genomic alteration that frequently appears in progressive disease after first- to third-generation EGFR-TKI treatment and is one of the acquired resistance mechanisms to EGFR-TKIs [12, 13, 16, 32]. In contrast to these previous studies, no MET amplification was detected in our study. The liquid biopsy of the NGS method used in this study cannot detect the genomic alternations of amplification, and 5 (16.1%) of the 18 (58.1%) study patients who underwent tissue biopsy for NGS testing had acquired alternations of amplification other than MET (ex. EGFR, CDK4 and FGFR). Therefore, the incidence rate of acquired MET amplification after the progression of osimertinib therapy may be underestimated, and tissue biopsy for NGS may be encouraged for the detection of acquired amplification leading to osimertinib resistance. Two patients (6.5%) in our study had MET alterations with point mutations detected by tissue biopsies.

Five patients in this study had BRAF mutations (3 with V600E mutations and 2 with non-V600E mutations), and previous studies reported BRAF mutations in EGFR-mutated NSCLC with resistance to EGFR-TKIs [33, 34]. For advanced BRAF-mutated NSCLC patients, patients with primary BRAF-V600E mutations respond to dabrafenib combined with trametinib therapy [35]. In this study, one patient had a KRAS-G12C mutation. KRAS-G12C is a driver mutation detected in human NSCLC, and drugs targeting KRAS-G12C have been developed and approved for the treatment of advanced NSCLC with KRAS-G12C mutations [36].

Some limitations in this study should be mentioned and clarified. First, osimertinib has been conditionally covered by national reimbursement in Taiwan since April 2020, and the benefits coverage are applicable only to first-line therapy in advanced NSCLC patients with EGFR exon 19 deletion mutation and brain metastasis and late-line therapy for T790M-mutated NSCLC patients experiencing acquired resistance to prior 1st- and 2nd-generation EGFR-TKI therapies [37]. The cost of first-line osimertinib therapy is not covered by Taiwanese national reimbursement for advanced NSCLC patients with primary L858R mutations, so few L858R-mutated NSCLC patients receive osimertinib as first-line therapy. To date, most untreated EGFR-mutated NSCLC patients in Taiwan have received 1st- and 2nd-generation EGFR-TKIs as first-line therapies [37, 38]. Osimertinib is more frequently used as a 2nd-line treatment for NSCLC patients with secondary T790M mutations after 1st- and 2nd-generation EGFR-TKIs than as a first-line treatment [37, 38]. Therefore, we recruited patients with the T790M mutation in this study, not those with primary common EGFR mutations (L858R and exon 19 deletion), who received first-line osimertinib. Second, although NGS via tissue or liquid biopsies is recommended for the clinical practice of NSCLC patient care, especially for the guidance of treatment options, the cost of NGS testing is higher than that of single-gene tests, so the cost-effectiveness of NGS remains a concern worldwide [39]. Given the limited funding of our study, the NGS test cannot be used extensively for NSCLC patients with acquired resistance to osimertinib. Taken together, these findings explained why our study recruited study patients with restricted criteria and had a small study population (31 patients and 18 who had paired liquid and tissue tests). All the study patients had T790M mutations that were either primary or acquired after prior first- or second-generation EGFR-TKI therapy. However, the genomic alteration profiles after acquired resistance to osimertinib may differ from those with common EGFR mutations (exon 19 deletion and L858R mutations) after front-line osimertinib therapy.

Although some genomic alternations with corresponding targeted drugs, including MET, BRAF-V600E, KRAS-G12C, ALK and RET inhibitors, were detected in this study, whether these patients received targeted therapies in their subsequent treatments was not recorded. Most patients presented acquired targetable driver mutations (ex. MET, BRAF-V600E, KRAS-G12C, ALK and RET) after osimertinib also had preserved EGFR mutations in both this study and previous studies [34, 40–44]. Several previous studies and case reports have demonstrated that the use of osimertinib in combination with drugs targeting these acquired driver genes is feasible and effective [40–44]. For example, a previous study reported that an NSCLC patient with acquired BRAF-V600E mutation resistance to osimertinib responded to combination therapy consisting of osimertinib, dabrafenib, and trametinib [41]. Another previous case report demonstrated that an advanced NSCLC patient whose EML4-ALK fusion was detected after acquired resistance to osimertinib therapy had a durable response to treatment with the ALK inhibitor ensartinib [43]. Taking the clinical experiences of previous studies and case reports together, more future studies investigating the efficacy of corresponding targeted therapies for genomic alterations after resistance to osimertinib are warranted. The therapeutic strategy of single targeted therapy and combination therapies may depend on the genomic profiles detected by NGS. In addition, given that acquired genomic alternations may be targetable after each line of targeted therapy, serial rebiopsies may be suggested to tailor the treatment strategy.

## Conclusion

NGS of either tissue or liquid biopsy samples from advanced T790M-mutated NSCLC patients with acquired resistance to osimertinib therapy revealed various genomic alterations. Tissue rebiopsy and liquid biopsy are complementary in the detection of additional resistance mutations but may provide distinct prognostic value in terms of T790M loss. Future studies focusing on subsequent tailored therapies on the basis of the results of NGS are warranted.

## Supplementary Information

Below is the link to the electronic supplementary material.

Supplementary file1 (DOCX 15 KB)

## Data Availability

The datasets generated and analyzed during this study are not publicly available because of local regulations regarding medical confidentiality.

## References

[1] Sharma SV, Bell DW, Settleman J, Haber DA. Epidermal growth factor receptor mutations in lung cancer. Nat Rev Cancer. 2007;7:169–81. 17318210 10.1038/nrc2088

[2] Zhou H, Geng F, Chen Y, Du J, Zhang X, Liu B, et al. The mineral dust-induced gene, mdig, regulates angiogenesis and lymphangiogenesis in lung adenocarcinoma by modulating the expression of VEGF-A/C/D via EGFR and HIF-1α signaling. Oncol Rep. 2021;45(5):60. 33760153 10.3892/or.2021.8011

[3] Liu C, Zheng S, Wang S, Wang X, Feng X, Sun N, et al. Development and external validation of a composite immune-clinical prognostic model associated with EGFR mutation in East-Asian patients with lung adenocarcinoma. Ther Adv Med Oncol. 2021;8(13):17588359211006948. 10.1177/17588359211006949 PMC8040386 33889215

[4] Yun CH, Mengwasser KE, Toms AV, Woo MS, Greulich H, Wong KK, et al. The T790M mutation in EGFR kinase causes drug resistance by increasing the affinity for ATP. Proc Natl Acad Sci U S A. 2008;105(6):2070–5. 18227510 10.1073/pnas.0709662105 PMC2538882

[5] Buder A, Hochmair MJ, Filipits M. The allele frequency of EGFR mutations predicts survival in advanced EGFR T790M-positive non-small cell lung cancer patients treated with osimertinib. Target Oncol. 2021;16(1):77–84. 33270169 10.1007/s11523-020-00781-3 PMC7810636

[6] Seto T, Nogami N, Yamamoto N, Atagi S, Tashiro N, Yoshimura Y, et al. Real-world EGFR T790M testing in advanced non-small-cell lung cancer: a prospective observational study in Japan. Oncol Ther. 2018;6(2):203–15. 32700028 10.1007/s40487-018-0064-8 PMC7359964

[7] Chang JW, Huang CY, Fang YF, Chang CF, Yang CT, Kuo CS, et al. Epidermal growth factor receptor tyrosine kinase inhibitors for de novo T790M mutation: a retrospective study of 44 patients. Thorac Cancer. 2022;13(13):1888–97. 35633141 10.1111/1759-7714.14272 PMC9250841

[8] McCoach CE, Jimeno A. Osimertinib, a third-generation tyrosine kinase inhibitor targeting non-small cell lung cancer with EGFR T790M mutations. Drugs Today (Barc). 2016;52(10):561–8. 27910964 10.1358/dot.2016.52.10.2541343

[9] Jänne PA, Yang JC, Kim DW, Planchard D, Ohe Y, Ramalingam SS, et al. AZD9291 in EGFR inhibitor-resistant non-small-cell lung cancer. N Engl J Med. 2015;372(18):1689–99. 25923549 10.1056/NEJMoa1411817

[10] Khozin S, Weinstock C, Blumenthal GM, Cheng J, He K, Zhuang L, et al. Osimertinib for the treatment of metastatic EGFR T790M mutation-positive non-small cell lung cancer. Clin Cancer Res. 2017;23(9):2131–5. 27923840 10.1158/1078-0432.CCR-16-1773

[11] Mok TS, Wu Y-L, Ahn M-J, Garassino MC, Kim HR, Ramalingam SS, et al. Osimertinib or platinum-pemetrexed in EGFR T790M-positive lung cancer. N Engl J Med. 2017;376(7):629–40. 27959700 10.1056/NEJMoa1612674 PMC6762027

[12] Oxnard GR, Hu Y, Mileham KF, Husain H, Costa DB, Tracy P, et al. Assessment of resistance mechanisms and clinical implications in patients with EGFR T790M-positive lung cancer and acquired resistance to osimertinib. JAMA Oncol. 2018;4(11):1527–34. 30073261 10.1001/jamaoncol.2018.2969 PMC6240476

[13] Roper N, Brown AL, Wei JS, Pack S, Trindade C, Kim C, et al. Clonal evolution and heterogeneity of osimertinib acquired resistance mechanisms in EGFR Mutant Lung Cancer. Cell Rep Med. 2020;1(1): 100007. 32483558 10.1016/j.xcrm.2020.100007 PMC7263628

[14] Chamorro DF, Cardona AF, Rodríguez J, Ruiz-Patiño A, Arrieta O, Moreno-Pérez DA, et al. Genomic landscape of primary resistance to osimertinib among hispanic patients with egfr-mutant non-small cell lung cancer (NSCLC): results of an observational longitudinal cohort study. Target Oncol. 2023;18(3):425–40. 37017806 10.1007/s11523-023-00955-9 PMC10192162

[15] Takamori S, Seto T, Yamaguchi M, Kinoshita F, Fujishita T, Ito K, et al. Case report: success of tepotinib therapy in overcoming resistance to osimertinib in a patient with EGFR-mutant lung adenocarcinoma with a potential acquired MET exon 14 skipping mutation. Front Oncol. 2022;13(12): 965741. 10.3389/fonc.2022.965741 PMC9608750 36313664

[16] Lee K, Kim D, Yoon S, Lee DH, Kim SW. Exploring the resistance mechanisms of second-line osimertinib and their prognostic implications using next-generation sequencing in patients with non-small-cell lung cancer. Eur J Cancer. 2021;148:202–10. 33744716 10.1016/j.ejca.2021.01.052

[17] Piotrowska Z, Niederst MJ, Karlovich CA, Wakelee HA, Neal JW, Mino-Kenudson M, et al. Heterogeneity underlies the emergence of EGFRT790 wild-type clones following treatment of T790M-positive cancers with a third-generation EGFR inhibitor. Cancer Discov. 2015;5(7):713–22. 25934077 10.1158/2159-8290.CD-15-0399 PMC4497836

[18] Oxnard GR, Thress KS, Alden RS, Lawrance R, Paweletz CP, Cantarini M, et al. Association between plasma genotyping and outcomes of treatment with osimertinib (AZD9291) in advanced non-small-cell lung cancer. J Clin Oncol. 2016;34(28):3375–82. 27354477 10.1200/JCO.2016.66.7162 PMC5035123

[19] Beagan JJ, Bach S, van Boerdonk RA, van Dijk E, Thunnissen E, van den Broek D, et al. Circulating tumor DNA analysis of EGFR-mutant non-small cell lung cancer patients receiving osimertinib following previous tyrosine kinase inhibitor treatment. Lung Cancer. 2020;145:173–80. 32460198 10.1016/j.lungcan.2020.04.039

[20] Cucchiara F, Del Re M, Valleggi S, Romei C, Petrini I, Lucchesi M, et al. Integrating liquid biopsy and radiomics to monitor clonal heterogeneity of EGFR-positive non-small cell lung cancer. Front Oncol. 2020;16(10): 593831. 10.3389/fonc.2020.593831 PMC7819134 33489892

[21] Tamiya A, Isa SI, Taniguchi Y, Nakagawa H, Atagi S, Ando M, et al. Prospective observational study of treatment resistance-related gene screening using plasma circulating tumor DNA in third-generation EGFR-TKI osimertinib therapy (Elucidator). Clin Lung Cancer. 2021;22(3):e336–41. 32641247 10.1016/j.cllc.2020.05.023

[22] Diaz LA Jr, Bardelli A. Liquid biopsies: genotyping circulating tumor DNA. J Clin Oncol. 2014;32(6):579–86. 24449238 10.1200/JCO.2012.45.2011 PMC4820760

[23] Mok T, Wu YL, Lee JS, Yu CJ, Sriuranpong V, Sandoval-Tan J, et al. Detection and dynamic changes of EGFR mutations from circulating tumor DNA as a predictor of survival outcomes in NSCLC patients treated with first-line intercalated erlotinib and chemotherapy. Clin Cancer Res. 2015;21(14):3196–203. 25829397 10.1158/1078-0432.CCR-14-2594

[24] Chiang CL, Huang HC, Shen CI, Luo YH, Chen YM, Chiu CH. Post-progression survival in secondary EGFR T790M-mutated non-small-cell lung cancer patients with and without osimertinib after failure of a previous EGFR TKI. Target Oncol. 2020;15(4):503–12. 32696212 10.1007/s11523-020-00737-7

[25] Kuo CS, Su PL, Wei YF, Ko JC, Tseng JS, Su J, et al. T790M detection rate after first-line combination therapy with bevacizumab and EGFR-TKIs in advanced NSCLC (TERRA study). Am J Cancer Res. 2023;13(7):3100–12. 37559987 PMC10408489

[26] Hsu PC, Chang JW, Chang CF, Huang CY, Yang CT, Kuo CS, et al. Sequential treatment in advanced non-small cell lung cancer harboring EGFR mutations. Ther Adv Respir Dis. 2022;16:17534666221132732. 36305280 10.1177/17534666221132731 PMC9619270

[27] Ortega-Granados AL, Artal-Cortes Á, Aguiar-Bujanda D, Oramas J, Fírvida JL, de Castro J, et al. Patterns of progression and feasibility of re-biopsy after first-line erlotinib for advanced EGFR mutation-positive non-small-cell lung cancer. Anticancer Res. 2019;39(3):1317–28. 30842164 10.21873/anticanres.13244

[28] Niederst MJ, Hu H, Mulvey HE, Lockerman EL, Garcia AR, Piotrowska Z, et al. The allelic context of the C797S mutation acquired upon treatment with third-generation EGFR inhibitors impacts sensitivity to subsequent treatment strategies. Clin Cancer Res. 2015;21(17):3924–33. 25964297 10.1158/1078-0432.CCR-15-0560 PMC4587765

[29] Arulananda S, Do H, Musafer A, Mitchell P, Dobrovic A, John T. Combination osimertinib and gefitinib in C797S and T790M EGFR-mutated non-small cell lung cancer. J Thorac Oncol. 2017;12(11):1728–32. 28843359 10.1016/j.jtho.2017.08.006

[30] Lu C, Wei XW, Wang Z, et al. Allelic context of EGFR C797X-mutant lung cancer defines four subtypes with heterogeneous genomic landscape and distinct clinical outcomes. J Thorac Oncol. 2024;19(4):601–12. 37981218 10.1016/j.jtho.2023.11.016

[31] Lu C, Wei XW, Wang Z, Zhou Z, Liu YT, Zheng D, et al. Potential utility of a 4th-generation EGFR-TKI and exploration of resistance mechanisms-an in vitro study. Biomedicines. 2024;12(7):1412. 39061985 10.3390/biomedicines12071412 PMC11273927

[32] Akli A, Girard N, Fallet V, Rousseau-Bussac G, Gounant V, Friard S, et al. Histomolecular resistance mechanisms to first-line osimertinib in EGFR-mutated advanced non-small cell lung cancer: a multicentric retrospective French study. Target Oncol. 2022;17(6):675–82. 36129569 10.1007/s11523-022-00915-9

[33] La Monica S, Minari R, Cretella D, Bonelli M, Fumarola C, Cavazzoni A, et al. Acquired BRAF G469A mutation as a resistance mechanism to first-line osimertinib treatment in NSCLC cell lines harboring an EGFR exon 19 deletion. Target Oncol. 2019;14(5):619–26. 31502118 10.1007/s11523-019-00669-x

[34] Morikawa K, Iinuma M, Shinozaki Y, Nishine H, Inoue T, Mineshita M. A case of advanced adenocarcinoma genetically confirmed with EGFR/BRAF co-mutation in both primary and metastatic lesions. Ther Adv Med Oncol. 2021;22(13):17588359211053420. 10.1177/17588359211053420 PMC8544759 34707694

[35] Yagami Y, Nakahara Y, Manabe H, Yamamoto H, Otani S, Sato T, et al. Promising response to dabrafenib plus trametinib in a patient with peritoneal carcinomatosis from non small lung cancer harboring BRAF V600E mutation. Onco Targets Ther. 2022;11(15):1369–74. 10.2147/OTT.S375246 PMC9664913 36388158

[36] Lee A. Sotorasib: a review in KRAS G12C mutation-positive non-small cell lung cancer. Target Oncol. 2022;17(6):727–33. 36315377 10.1007/s11523-022-00922-w PMC9715446

[37] Hsu PC, Lee SH, Chiu LC, Lee CS, Wu CE, Kuo SC, et al. Afatinib in untreated stage IIIB/IV lung adenocarcinoma with major uncommon epidermal growth factor receptor (EGFR) mutations (G719X/L861Q/S768I): a multicenter observational study in Taiwan. Target Oncol. 2023;18(2):195–207. 36805452 10.1007/s11523-023-00946-w PMC10042759

[38] Hsu PC, Huang CY, Lin YC, Lee SH, Chiu LC, Wu CE, et al. Sequential treatment in advanced epidermal growth factor receptor-mutated lung adenocarcinoma patients receiving first-line bevacizumab combined with 1st/2nd-generation EGFR-tyrosine kinase inhibitors. Front Oncol. 2023;3(13):1249106. 10.3389/fonc.2023.1249106 PMC10579797 37854677

[39] Kohara T, Ikeda S, Ishikawa KB. Cost-effectiveness analysis of the Oncomine™ Dx target test MultiCDx system using next-generation sequencing and single-gene test in advanced and recurrent nonsquamous non-small-cell lung cancer. JMA J. 2024;7(3):375–86. 39114611 10.31662/jmaj.2023-0206 PMC11301018

[40] Cho BC, Kim DW, Spira AI, Gomez JE, Haura EB, Kim SW, et al. Amivantamab plus lazertinib in osimertinib-relapsed EGFR-mutant advanced non-small cell lung cancer: a phase 1 trial. Nat Med. 2023;29(10):2577–85. 37710001 10.1038/s41591-023-02554-7 PMC10579096

[41] Huang Y, Gan J, Guo K, Deng Y, Fang W. Acquired BRAF V600E mutation mediated resistance to osimertinib and responded to osimertinib, dabrafenib, and trametinib combination therapy. J Thorac Oncol. 2019;14(10):e236–7. 31558239 10.1016/j.jtho.2019.05.040

[42] Ernst SM, Uzun S, Paats MS, van Marion R, Atmodimedjo PN, de Jonge E, et al. Efficacy and tolerability of osimertinib and sotorasib combination treatment for osimertinib resistance caused by KRAS G12C mutation: a report of two cases. JCO Precis Oncol. 2023;7: e2300451. 38096473 10.1200/PO.23.00451 PMC10735074

[43] Guo Y, Zhang R, Meng Y, Wang L, Zheng L, You J. Case report: durable response of ensartinib targeting EML4-ALK fusion in osimertinib-resistant non-small cell lung cancer. Front Pharmacol. 2024;29(15):1359403. 10.3389/fphar.2024.1359403 PMC11317239 39135785

[44] Rotow J, Patel JD, Hanley MP, Yu H, Awad M, Goldman JW, et al. Osimertinib and selpercatinib efficacy, safety, and resistance in a multicenter, prospectively treated cohort of EGFR-mutant and RET fusion-positive lung cancers. Clin Cancer Res. 2023;29(16):2979–87. 36996322 10.1158/1078-0432.CCR-22-2189 PMC10524391

