# Supplementary material for: Analysis of genomic alternations in epidermal growth factor receptor (EGFR)-T790M-mutated non-small cell lung cancer (NSCLC) patients with acquired resistance to osimertinib therapy
Source: Clin Transl Oncol. 2024 Sep 24;27(5):1967–79. doi: 10.1007/s12094-024-03727-7 (PMC12033095; doi:10.1007/s12094-024-03727-7)
Supplement: Supplementary file 1 — Supplementary file1 (DOCX 15 KB) [file 12094_2024_3727_MOESM1_ESM.docx]

| InDel genes | | | CNVs | | Fusions | |
| --- | --- | --- | --- | --- | --- | --- |
| AKT1 | FGFR2 | MAP2K1 | ALK | KIT | ABL1 | FGFR2 |
| ALK | FGFR3 | MAP2K2 | AR | KRAS | AKT3 | FGFR3 |
| AR | GNA11 | MET | BRAF | MET | ALK | MET |
| BRAF | GNAQ | MTOR | CCND1 | MYC | AXL | NTRK1 |
| CDK4 | HRAS | NRAS | CDK4 | MYCN | BRAF | NTRK2 |
| CTNNB1 | IDH1 | PDGFRA | CDK6 | PDGFRA | EGFR | NTRK3 |
| DDR2 | IDH2 | PIK3CA | EGFR | PIK3CA | ERBB2 | PDGFRA |
| EGFR | JAK1 | RAF1 | ERBB2 |  | ERG | PPARG |
| ERBB2 | JAK2 | RET | FGFR1 |  | ETV1 | RAF1 |
| ERBB3 | JAK3 | ROS1 | FGFR2 |  | ETV4 | RET |
| ERBB4 | KIT | SMO | FGFR3 |  | ETV5 | ROS1 |
| ESR1 | KRAS |  | FGFR4 |  | FGFR1 |  |

**Supplementary Table S1**. List of targeted genes
